# Emerging triple-reassortant influenza C virus with household-associated infection during an influenza A(H3N2) outbreak, China, 2022

**DOI:** 10.1080/22221751.2023.2175593

**Published:** 2023-03-07

**Authors:** Lan Cao, Ying Lu, Chaojun Xie, Yiyun Chen, Lijun Liang, Tengfei Zhou, Ziyi Zeng, Chen Wen, Biao Di, Baisheng Li, Kuibiao Li, Zhoubin Zhang

**Affiliations:** a Institute of Public Health, Guangzhou Medical University and Guangzhou Center for Disease Control and Prevention, Guangzhou, People’s Republic of China; b Huadu District Center for Disease Control and Prevention, Guangzhou, People’s Republic of China; c Guangdong Provincial Center for Disease Control and Prevention, Guangzhou, People’s Republic of China

**Keywords:** Influenza C virus, China, family clustering, infection, triple reassortant

## Abstract

Recent research have shown that influenza C virus (ICV) has a possible higher clinical impact than previously thought. But knowledge about ICV is limited compared with influenza A and B viruses, due to poor systematic surveillance and inability to propagate. Herein, a case infected with triple reassortant ICV was identified during an influenza A(H3N2) outbreak, which was the first report of ICV infection in mainland China. Phylogenetic analysis showed that this ICV was triple reassortant. Serological evidence revealed that the index case might be related to family-clustering infection. Therefore, it is essential to heighten surveillance for the prevalence and variation of ICV in China, during the COVID-19 pandemic.

The annual epidemics of seasonal influenza have caused numerous deaths, high incidence rate, and huge economic losses [1], which are caused by the co-circulation of influenza A and B viruses in different proportions [2]. Influenza C virus was first isolated in 1947 in the United States [3]. For a long time, the knowledge about ICV was limited due to poor systematic surveillance and inability to propagate, although seroprevalence of ICV has been shown high positive rates, with the virus being distributed worldwide in the human population and initial exposure or infection occurring during childhood [4]. Recently, studies have found that ICV could be associated with community-acquired pneumonia and severe disease with lower respiratory tract infections in children, which had higher clinical impact in specified patient populations than previously thought [5]. However, the knowledge of epidemiologic and genetic characteristics of ICV was still unclear, especially in mainland China.

During the COVID-19 pandemic, the influenza epidemic increased in China and caused the influenza outbreak in 2021–2022 [2]. Since May 2022, influenza A(H3N2) viruses continued to prevail in China. On 14 June, 2022, there was an abnormal increase in influenza-like illness in a kindergarten in Guangzhou, involving 68 children aged from 3 to 4. Oropharyngeal swabs were sampled on the next day. Viral RNAs were extracted by QIAamp Viral RNA Mini Kit (Qiagen, Dusseldorf, Germany). Influenza A, B, and C viruses were detected using qRT-PCR kits (BioGerm, Shanghai, China). Results showed that four samples tested positive for the influenza A(H3N2) virus and one tested positive for the influenza C virus (Cycle of threshold: 33).

The index case infected with ICV was a 4-year-old girl, with no injection of influenza vaccine, who had symptoms, including runny nose, stuffy nose, slight cough, pharyngeal congestion, breathing sound aggravated, and conjunctival congestion, no vomiting and diarrhoea. She got fever for 6 days intermittently, with the highest temperature of 38.7°C on June 14. She was diagnosed with acute upper respiratory tract infection and conjunctivitis in Community Hospital Center. The girl took Paracetamol Pseudoephedrine Hydrochloride and Chlorphenamine Maleate Tablets, Levofloxacin, and oseltamivir during treatment and got well soon.

To further identify the causative pathogen, we performed next-generation-sequencing (NGS), virus isolation, and serological test. Viral whole-genome were amplified as previously described [6]. Concurrently, next-generation targeted sequencing of ICV genomes was conducted on Illumina MiniSeq, with Sequencing Library Prep kits of Illumina Nextera® XT (Illumina, San Diego, USA) and MiniSeq Rapid Reagent Kit (100 cycles) (Illumina, San Diego, USA). Raw fastq data was analysed by QIAGEN® CLC Genomics Workbench Software (version 22). The seven segmented genes were edited with Lasergene (version 7.0) and analysed with the MEGA Software (version 6.0). Phylogenetic trees of the seven genes were constructed using the neighbour-joining method, with *p*-distance as a substitution model, and 1000 bootstrapped replicates together with previously reported sequences [7] downloaded from the EpiFlu Database of GISAID (www.gisaid.org). Previous studies have shown that HE genes of ICV fall into six genetic lineages [8] that correspond to antigenic clusters [1]. Neighbour-joining trees showed that this virus named C/Guangzhou/05166/2022 was triple reassortant, with the gene of HE belonged to KA176-like lineage, the genes of PB2, PB1, M, and NS belonged to PB11581-like lineage, the genes of P3 and NP belonged to MS80-like lineage (Figure 1). Phylogenetic analysis demonstrated that the genotype of C/Guangzhou/05166/2022 was different from the prevalent strains isolated from Hong Kong (2015–2020) and Japan Philippines (2009–2013) [6,7], though the HE gene belongs to the same evolutionary branch of KA176 as the Hong Kong epidemic strain. This similar genotype strain could be traced back to the C/Miyagi/9/96 and C/Saitama/3/2000 of Japanese epidemic strains from 1996 to 2000, which revealed that the diversity of ICV genes pool in Asia provided conditions that various gene segments could be mixed and reassorted. Figure 1.Phylogenetic trees for seven segments of the influenza C virus. The nucleotide sequences of HE, M, NP, NS, P3, PB1, and PB2 genes of influenza C virus were used for analysis. The isolated C/Guangzhou/05166/2022 strain is marked with red circles.

**Figure:**
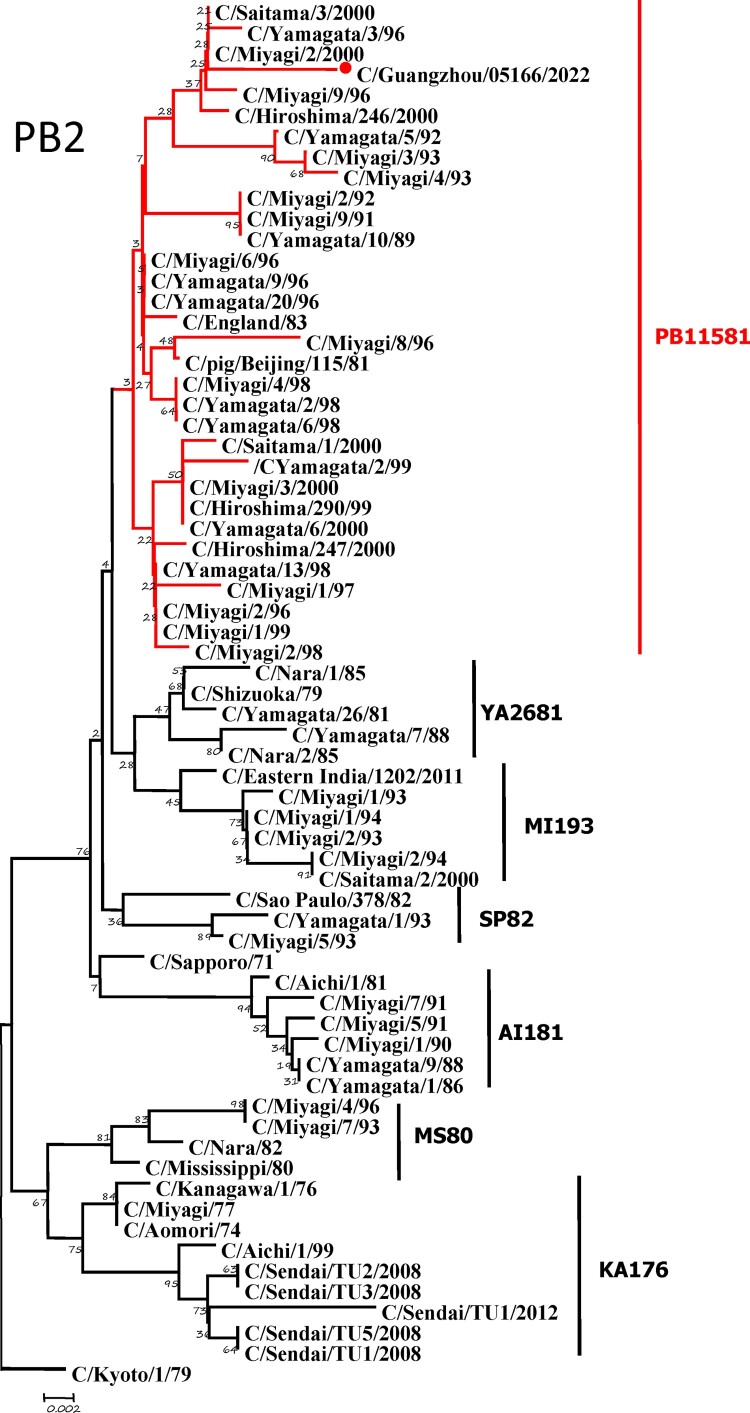

**Figure:**
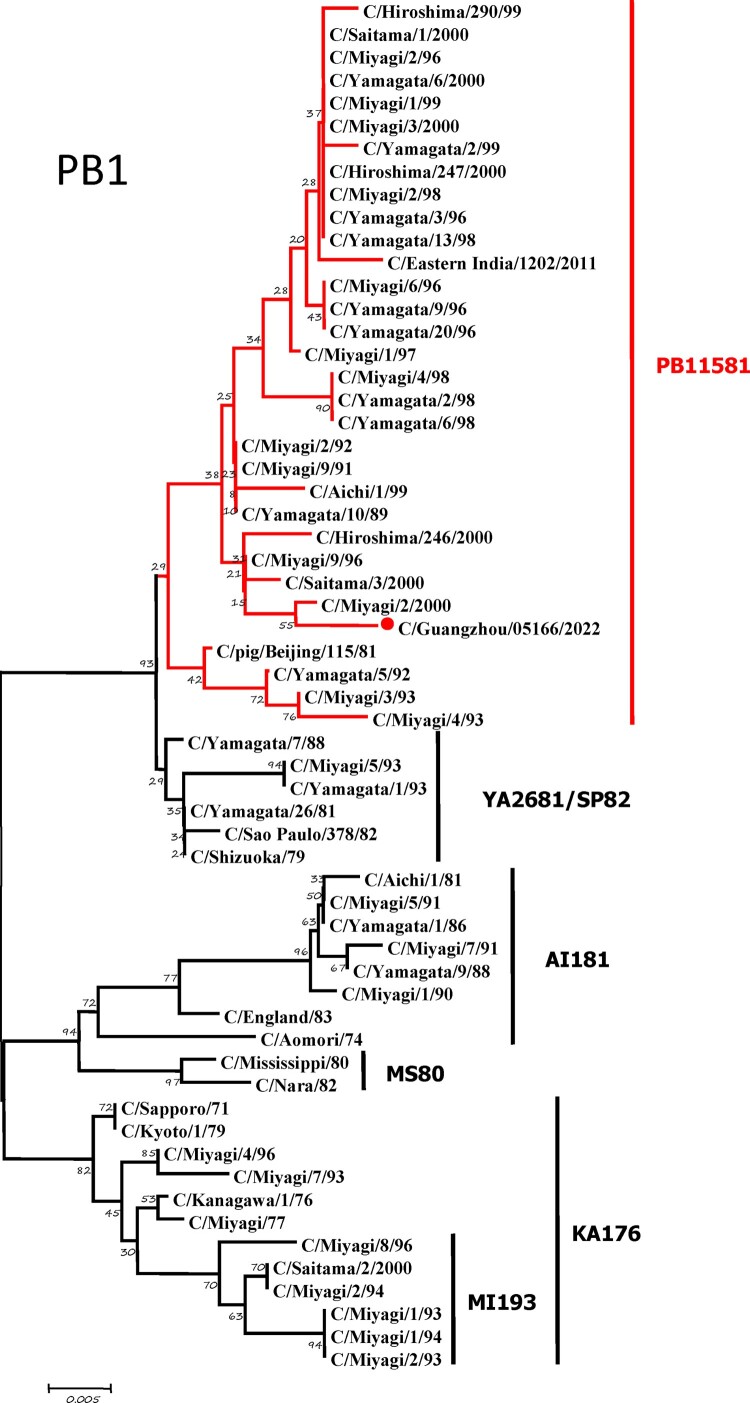

**Figure:**
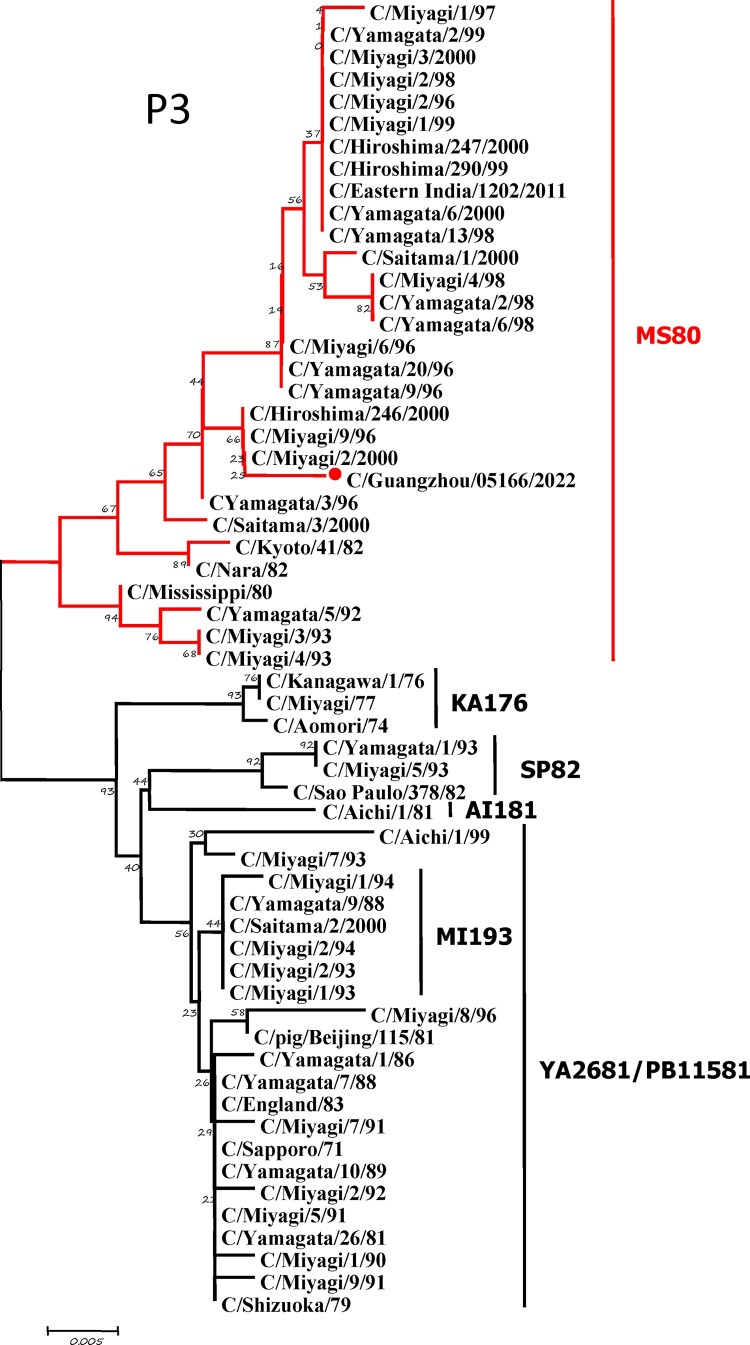

**Figure:**
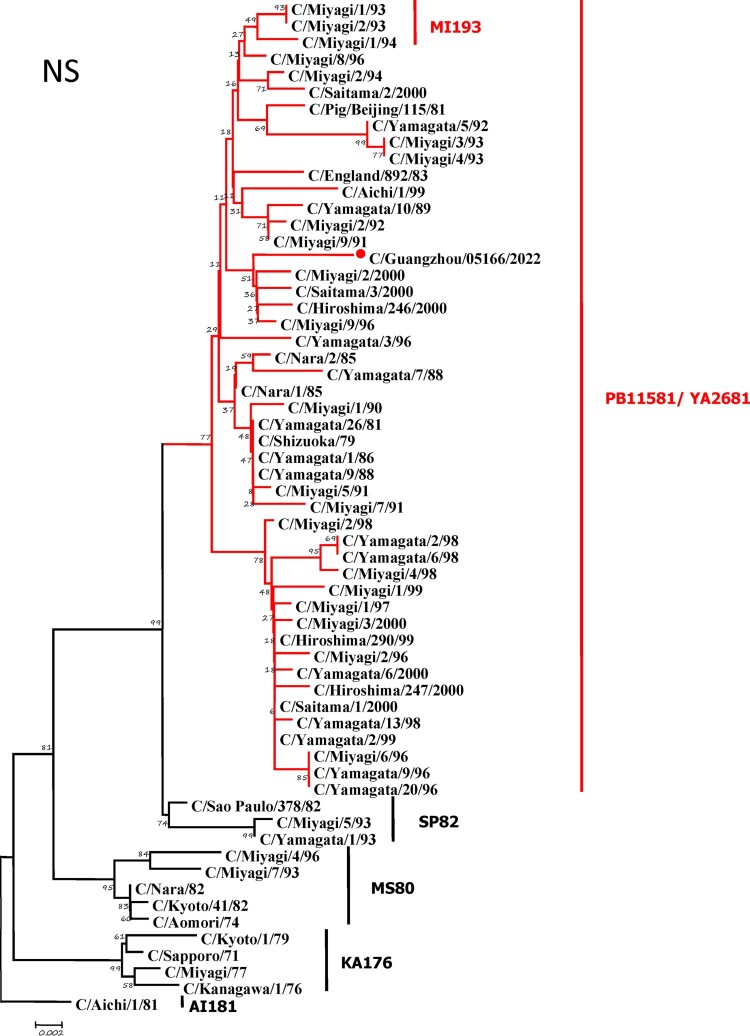

**Figure:**
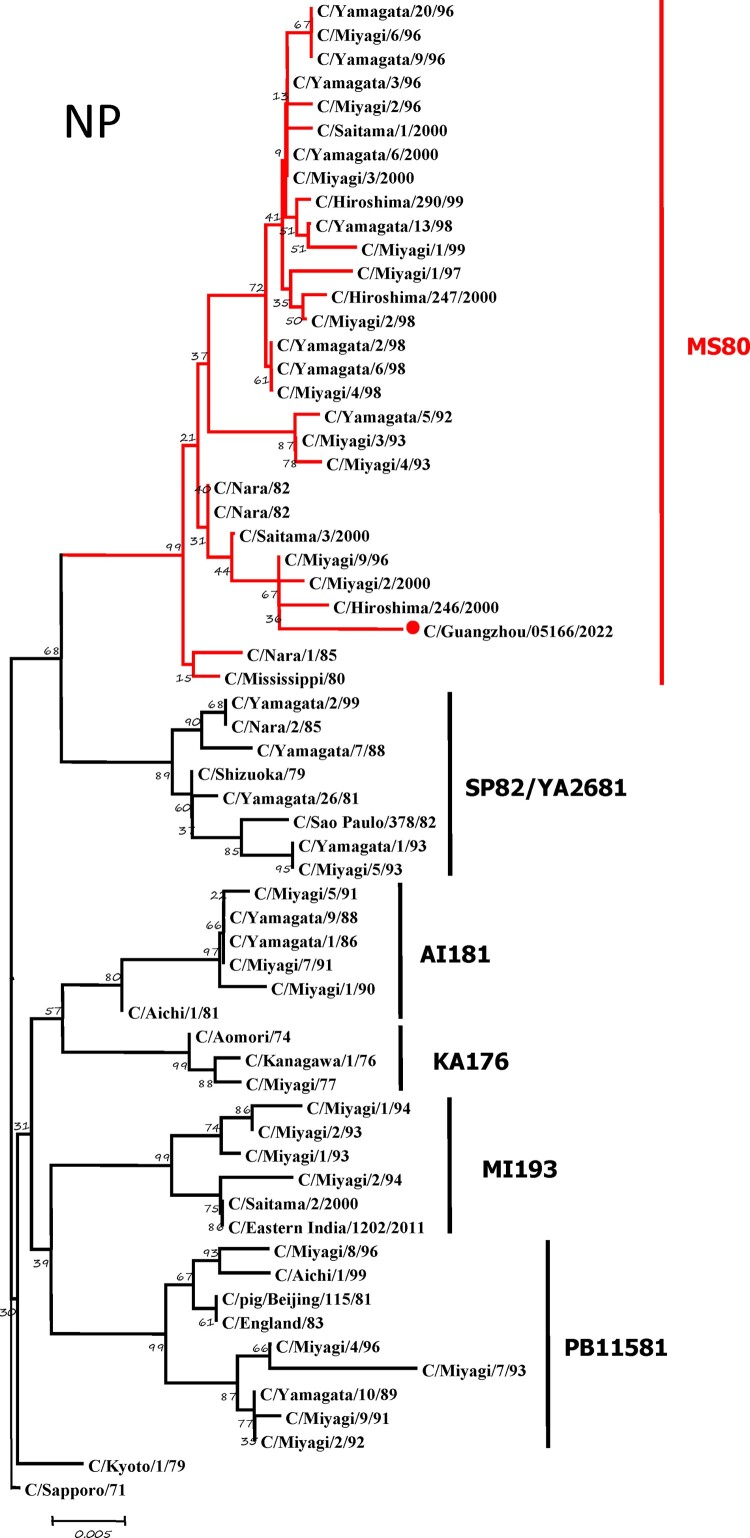

**Figure:**
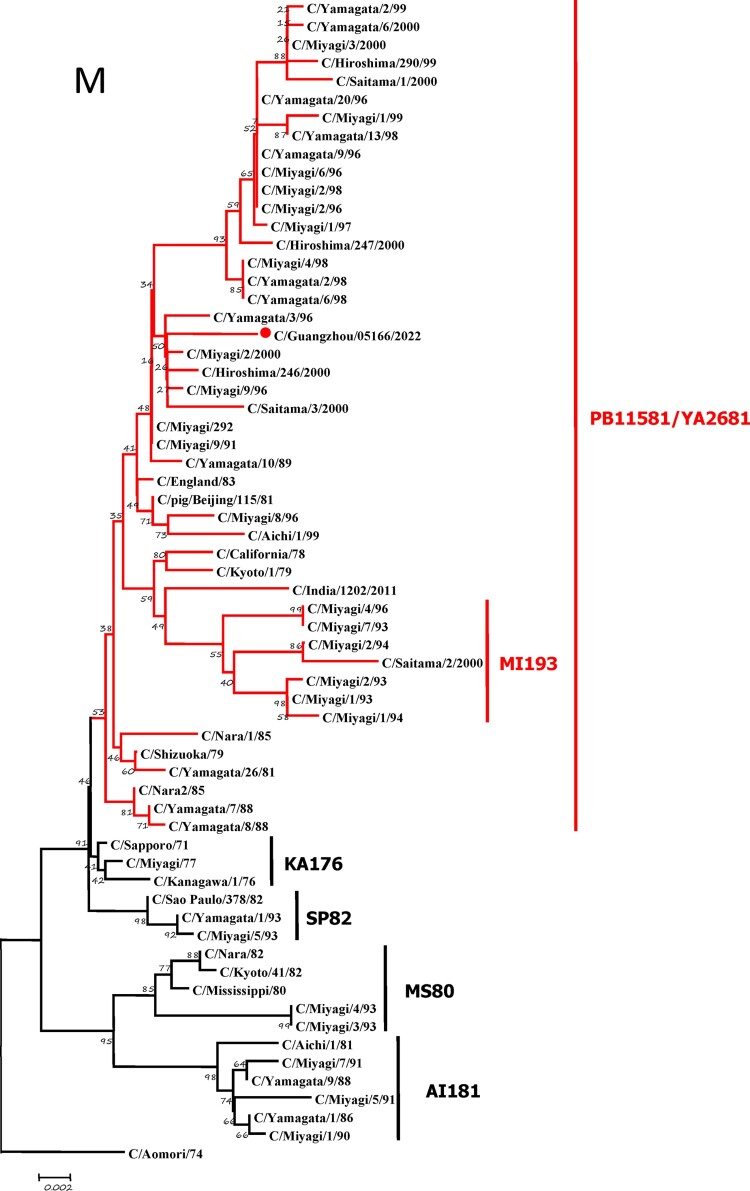

**Figure:**
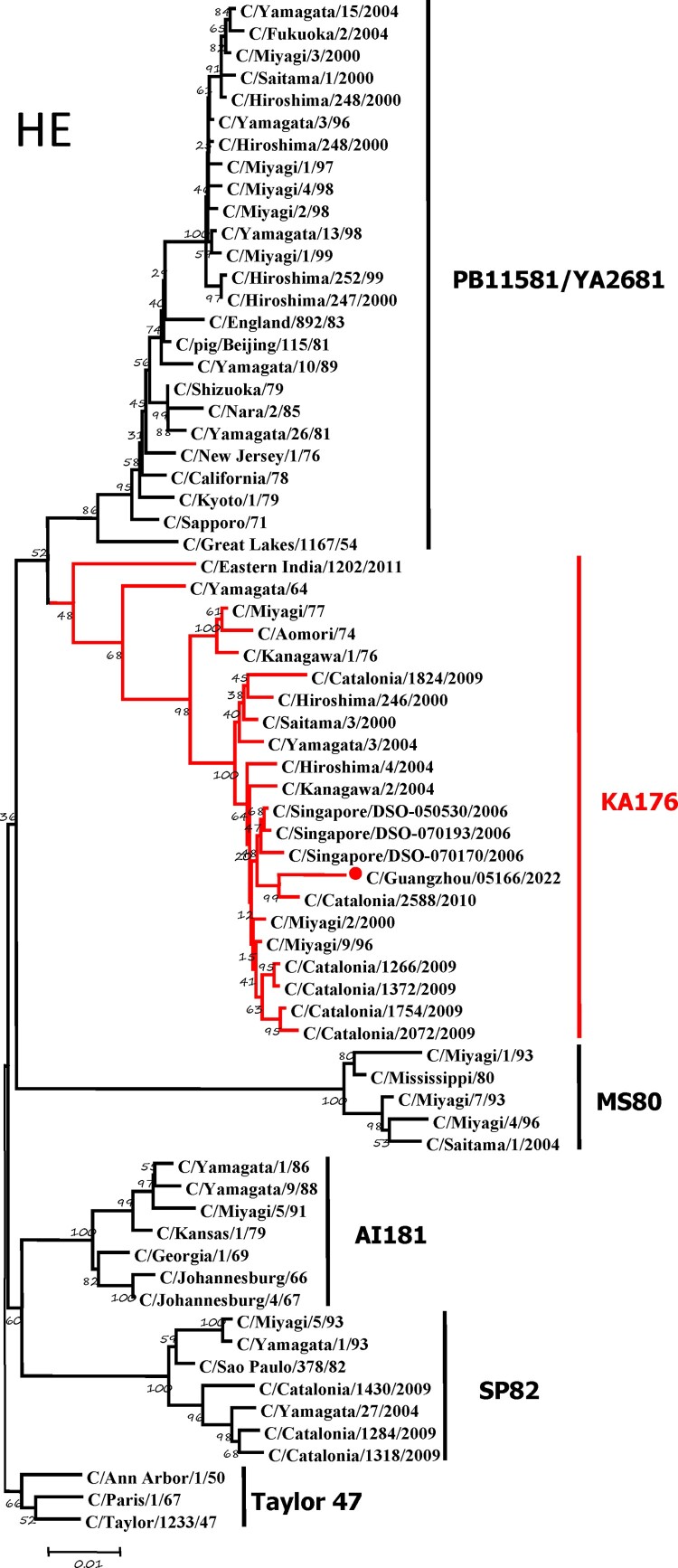

C/pig/Beijing/115/81, the representative strains of PB11581-like lineage, was isolated from a pig in 1981 in China [9], which proved that ICV could be transmitted between different species. In this study, the genes of PB2, PB1, M, and NS belonged to PB11581-like lineage, which was speculated that the homologous swine-derived gene pools might continue to prevail in China. Since influenza A viruses were prone to be reassorted, such as H1N1(2009pdm) virus was evolved from the recombinant zoonotic event involving pigs [10], the possibility of ICV reassortment occurrence between human and swine didn’t be ignored. Therefore it was necessary to monitor the prevalence and recurrence of multiple ICV lineages which could induce cross-species transmission and pose potential zoonotic and pandemic threats by reassortment [11].

The index case’s serum samples were collected during acute and convalescence phases (on June 18 and on July 4, respectively) and the serum samples of her family also were collected (on July 30), which were designed to trace the infective source by serological retrospective detection. Antibody diagnosis showed that the Hemagglutination Inhibition titre of convalescence-phase serum sample of index cases was 1:640, which was much higher than that of acute-phase (less than 1:10) against the strain C/Guangzhou/05166E2/2022 isolated in this study, which could further confirm that the index case was infected with ICV. Notably, index case’s mother and grandmother also had positive antibody conversion (Hemagglutination Inhibition titres 1:320 and 1:640, respectively), which could prompt their co-exposure to infection with ICV in the family currently.

The propagation of ICV to a high hemagglutination titre has been proved difficult [12]. We attempted to isolate ICV in the allantoic cavity of 9-day-old SPF embryonated eggs and Madin–Darby canine kidney (MDCK) cell culture from all oropharyngeal swabs collected. The isolations in the MDCK cell culture were unsuccessful. But fortunately, the strain C/Guangzhou/05166E2/2022 was successfully isolated from embryonated eggs and obtained stably high titre during passages, which could provide important material way for epidemiological investigation and virological research, such as retrospective serological investigation, interactions between ICV and hosts, and across-species transmission in future.

The long-term surveillance of ICV infections revealed that biennial epidemic waves occurred both in Hong Kong [13] and Japan [7], with the detection rate of 0.22% being associated with outbreaks in the winters in Hong Kong [6]. Research have pointed out that ICV could also cause lower respiratory infections, such as bronchitis and pneumonia, which might have significant clinical impacts in human [14]. However, the prevalence baseline of ICV was still unknown in mainland China. Thus it was necessary to carry out epidemiological surveillance of influenza C during the influenza season.

With the continuous evolution of SARS-Cov-2, the proportion of mild and asymptomatic COVID-19 cases is relatively high, with similar symptoms as influenza C infection, so it is difficult to distinguish clinically. Experts have presented that the superposition effect of influenza and COVID-19 co-epidemic should be alert in this winter of 2022. We suggest that the epidemic impact of influenza C should be monitored during the COVID-19 pandemic in China.

## Data Availability

The full-genome sequences of C/Guangzhou/05166E2/2022 have been deposited in GISAID under the accession number EPI2110306-EPI2110312.
